# A conversation with Steven Pavletic, MD, MS, senior clinician, National Cancer Institute, Center for Cancer Research

**DOI:** 10.1017/cts.2023.29

**Published:** 2023-04-04

**Authors:** Kathy Siranosian

**Affiliations:** Clinical Research Forum, Washington, DC, USA

## Top 10 Clinical Research Achievement Awards Q & A

This article is part of a series of interviews with recipients of Clinical Research Forum’s Top 10 Clinical Research Achievement Awards. This article is with Steven Pavletic, MD, MS, Senior Clinician, National Cancer Institute, Center for Cancer Research. Dr Pavletic and his co-investigators study chronic graft-versus-host disease (cGVHD), the most serious late complication after allogeneic hematopoietic stem cell transplantation (HSCT). Dr Pavletic received a 2022 Top 10 Clinical Research Achievement Award for the development of pomalidomide in the treatment of chronic graft-versus-host disease [1]. *The interview has been edited for length and clarity.*

## What first got you interested in a career in clinical research?

I received my MD and internal medicine training from the University of Zagreb School of Medicine in Croatia. Then, I completed a clinical fellowship in bone marrow transplantation at the Fred Hutchinson Cancer Research Center in Seattle, WA. Dr Don Thomas, who is known as the father of bone marrow transplantation, won the Nobel Prize my first year there and working in that environment was a real turning point for me. I realized how medical developments rely on clinical research and that if I wanted to be on the cutting edge, I needed to be involved in the research community.

## Where did your career path take you next?

From Fred Hutch, I went on to residency and a hematology and oncology fellowship at the University of Nebraska Medical Center (UNMC) in Omaha, NE, where I then served as the Director of the Allogeneic Stem Cell Transplantation Program. In 2002, I received an appointment at the National Cancer Institute (NCI) and an adjunct appointment at the National Institute for Arthritis and Musculoskeletal and Skin Diseases at NIH. Now, I’m directing the NIH Chronic Graft-versus-host Disease (cGVHD) Study Group, practicing medicine and oncology and researching ways to cure blood cancers and treat cGVHD.

## When did you start working on the clinical trial to determine the safety, efficacy, and preferred dose of pomaliodomide for certain people with cGVHD?

In clinical research, progress is incremental. When I was in Seattle and Nebraska, we understood that bone marrow transplantation can cure patients with leukemia, but we also knew about potential complications, including graft-versus-host disease. Essentially, it’s a disease that happens when donor cells attack the recipient. Treatments for blood cancers have evolved and now we also use stem cells for transplants, but with the growing numbers of patients receiving them, we’re seeing chronic graft-versus-host disease. Patients get cured from cancer, but then can be disabled for life from that complication. Since chronic graft-versus-host disease is a new disease in medicine, there is still much to be learned about why it happens and how to prevent it. Our clinical team to study this came together in 2002. At that time, there was no clinical research or trials because there were no tools to study or diagnose chronic graft-versus-host disease. Gradually, we developed methods to conduct clinical trials and measure response to treatments in a standardized way. So it took about 20 years to get us to the point where we are today and able to complete trials of new agents in cGVHD.

## How do you stay motivated when it can take decades for clinical research results?

That’s a great question. I think you have to what could be called a fire in the belly, a desire to know the answer and to ultimately help patients. It’s also very motivating to work with great teams – these are the people who help you keep going, pushing ahead to the ultimate goal. I always try to take advantage of opportunities to stay inspired and keep learning. A well-designed trial will have milestones along the way and even a negative result is its own success because it teaches you something. Over a project that takes years, bringing in junior people can be very motivating, as well. They provide new energy and inspiration.

## Which research questions are you tackling now?

There have been a handful of critical clinical studies that have concluded over the past few years, so we are making good progress in understanding chronic graft-versus-host disease. Unfortunately, though, there are still too many patients that are not having sufficient responses to the available treatments. One area where we can improve is in learning how to predict who’s going to respond to certain drugs. That way we can avoid unnecessary treatments and perhaps introduce novel combinations of new drugs that are less toxic. This type of more personalized medicine is where we are repositioning now.

## What will it take to achieve these new therapy options?

Success will take a broad, community effort with academia, industry, regulatory, and patient advocates all at the table working together. Progress requires this kind of collaboration by people of different backgrounds. Recently, I heard someone at a conference say that “the days of the rock start are over” and that we are now living in “the days of the orchestra.” I really believe that. The way to achieve new therapies is by bringing together individuals from different specialties and backgrounds, all working towards the same goal. We’ve been very successful with our interdisciplinary approach, and it’s a true team effort that’s very collegial and constructive. Also, I cannot emphasize enough how important robust public funding is in propelling clinical trials, particularly around innovation.

## Looking back at what you’ve learned over the years, what advice do you have for a clinical researcher starting out now?

One of the best pieces of advice I have is to follow your heart. When you’re working in clinical research, you can't expect immediate gratification or even immediate credit, but those things will come if you keep doing what you enjoy. You also have to realize the importance of working as part of a team. I was 10 or 15 years into my career when I realized how much I enjoy building teams, working with them, and making them successful. It comes down to staying focused on the ultimate outcome. There may be failures along the way but they’re just part of the progress. What really counts is what you learn and how you keep moving forward. Lastly, get the best possible education and training you can. Learn from great examples. Then, go on and do something great yourself.
